# Alternative splicing factors and cardiac disease: more than just missplicing?

**DOI:** 10.1261/rna.080332.124

**Published:** 2025-03

**Authors:** Zachery R. Gregorich, Wei Guo

**Affiliations:** Department of Animal and Dairy Sciences, University of Wisconsin-Madison, Madison, Wisconsin 53706, USA

**Keywords:** alternative splicing, cardiomyopathy, RBM20, RNA granules

## Abstract

Alternative splicing (AS) is the process wherein the exons from a single gene are joined in different combinations to produce nonidentical, albeit related, RNA transcripts. This process is important for the development and physiological function of many organs and is particularly important in the heart. Notably, AS has been implicated in cardiac disease and failure, and a growing number of genetic variants in AS factors have been identified in association with cardiac malformation and/or disease. With the field poised to interrogate how these variants affect cardiac development and disease, an understandable point of emphasis will undoubtedly be on downstream target gene missplicing. In this Perspective article, we would like to encourage consideration not only of the potential for novel disease mechanisms, but also for contributions from disruption of the ever-expanding list of nonsplicing functions ascribed to many AS factors. We discuss the emergence of a novel cardiac disease mechanism based on pathogenic RNA granules and speculate on the generality of such a mechanism among localization-disrupting AS factor genetic variants. We also highlight emerging nonsplicing functions attributed to several AS factors with cardiac disease-associated genetic variants in the hopes of pointing to avenues for exploration of mechanisms that may contribute to disease alongside target gene missplicing.

## INTRODUCTION

Alternative splicing (AS) is an important posttranscriptional mechanism that plays key roles in generating protein diversity (Nilsen and Graveley 2010). It is estimated that over 95% of multiexon genes are regulated via AS (Pan et al. 2008; Wang et al. 2008). Additionally, AS can regulate gene expression through nonsense-mediated decay (Lewis et al. 2003). It has been found that AS is important not only for coding genes but also for long noncoding RNAs as well (Deveson et al. 2018). For an overview of regulatory mechanisms in AS, the reader is referred to the many excellent reviews published on this topic (Licatalosi and Darnell 2010; Irimia and Blencowe 2012; Kornblihtt et al. 2013; Fu and Ares 2014; Baralle and Giudice 2017; Kastner et al. 2019; Shenasa and Hertel 2019; Ule and Blencowe 2019; Gordon et al. 2021). AS is crucial for the development of multiple organs but contributes the greatest to the development of the brain and striated muscles, including cardiac muscle (Wang et al. 2008). In fact, cardiac genes, such as *TNNT2*, which encodes the cardiac-specific isoform of troponin T, were among the first found to be regulated by this process (Medford et al. 1984). Subsequent transcriptome-wide studies have revealed that broad and dramatic changes in protein isoform expression, brought about through AS, are crucial for the development of the heart (Kalsotra et al. 2008; Giudice et al. 2014). This importance is further underscored by the fact that the genetic ablation of various AS factors in the hearts of rodents either manifests or predisposes the heart to dysfunction and/or failure, as reviewed elsewhere (Montañés-Agudo et al. 2023; Cao et al. 2024).

Given the important role that AS plays in the development and function of the heart, it is perhaps not surprising that this process has also been implicated in cardiac disease and failure (Kong et al. 2010; Lee et al. 2011; Song et al. 2012). Studies have shown that certain AS factors are differentially expressed in various cardiomyopathies, as well as in heart failure, with a recent example being SLM2, which is upregulated in dilated cardiomyopathy (DCM) (Boeckel et al. 2022). Furthermore, a growing number of genetic variants in AS factors have been identified in human patients in association with cardiac malformation and/or disease (Brauch et al. 2009; Johnston et al. 2010; Homsy et al. 2015; Wang et al. 2016; Au et al. 2018; Yu et al. 2018; Chen et al. 2024); and this number will undoubtedly continue to rise as high-throughput sequencing becomes increasingly affordable and achieves greater use in clinical diagnostics (Pasche and Absher 2011).

When seeking to establish the molecular mechanisms associated with disease-causing genetic variants in AS factors, a logical first step is to focus on the role that downstream target gene missplicing plays in pathobiology as exemplified by our own studies (Guo et al. 2012). While this is an understandable and likely fruitful place to begin, it is important to keep in mind that this may be only one part of the equation. To exemplify this, we provide a brief overview of our evolving understanding of the molecular mechanisms in cardiac disease caused by pathogenic genetic variants in the AS factor RNA-binding motif protein 20 (RBM20), often referred to as RBM20 cardiomyopathy (Gregorich et al. 2024). We highlight the identification of pathogenic RBM20 granules as a causative agent in cardiac disease and discuss whether such a mechanism could be shared among localization-disrupting variants in AS factors. We conclude by pointing out several additional nonsplicing functions of AS factors with genetic variants implicated in cardiac malformation and disease, beginning with RBM20. Importantly, the purpose here is not to argue that target gene missplicing downstream from AS factor variants does not contribute to cardiac pathobiology and disease. Instead, we wish to encourage investigation of other mechanisms and processes that may also be involved.

### Pathogenic cytoplasmic granules and missplicing: dual contributors to RBM20 cardiomyopathy

Loss of the *Rbm20* gene, which encodes the muscle-specific splicing factor RBM20, was identified by our laboratory over a decade ago as the cause of a DCM-like phenotype in a rat strain deficient in splicing of the giant sarcomeric protein titin (Guo et al. 2012). We immediately recognized that the phenotype in *Rbm20* knockout (KO) rats resembled, at least in some respects, that previously reported in individuals with DCM caused by pathogenic genetic variants in *RBM20* with cardiac remodeling, increased susceptibility to arrhythmia, and premature mortality (Brauch et al. 2009). Beyond titin, this study and others identified approximately 30 other genes subject to AS regulated by RBM20 (Guo et al. 2012; Maatz et al. 2014). Critically, these included several genes important for the regulation of Ca^2+^-handling, such as *Camk2d*, *Ryr2*, and *Trdn* (Guo et al. 2012; Maatz et al. 2014). Hence, a putative mechanistic basis for RBM20 cardiomyopathy was established based on target gene missplicing. Specifically, this disease was presumed to arise because of (1) reduced ventricular wall tension resulting from altered titin splicing and (2) impaired contractility secondary to changes in the splicing of genes important for Ca^2+^-handling (Guo et al. 2012, 2018, 2021; Linke and Bücker 2012; Methawasin et al. 2014). This splicing-centric mechanism received further support with the demonstration that *Rbm20* KO mice phenocopy KO rats with DCM and proarrhythmic changes expected to underlie the propensity for arrhythmias in individuals carrying pathogenic *RBM20* genetic variants (van den Hoogenhof et al. 2018).

Yet, it quickly became apparent that not all data supported target gene missplicing as the sole mechanism of disease pathogenesis in RBM20 cardiomyopathy. Firstly, there was a pronounced phenotypic discrepancy between *Rbm20* KO rodents and patients carrying pathogenic genetic variants in *RBM20*, with the latter exhibiting a much more severe phenotype. To illustrate, even homozygous *Rbm20* KO rats do not exhibit signs of systolic dysfunction and approximately 83% of rats live over 18 months without developing heart failure (Guo et al. 2012). Conversely, all reported human carriers of pathogenic genetic variants in *RBM20* are heterozygous and most develop severe disease, as evidenced by the requirement for heart transplantation at earlier age in *RBM20* variant carriers compared to that in carriers of pathogenic genetic variants in other DCM-associated genes (Kayvanpour et al. 2017). Secondly, mice expressing RBM20 lacking the RRM, which renders the protein splicing deficient, did not develop DCM despite similar missplicing of major RBM20 target genes like *Ttn* and *Camk2d* (Methawasin et al. 2014). This lack of phenotype received further support recently with the generation of mice expressing a variant of uncertain significance (I536T) in the RRM of RBM20, previously identified in a case of sudden cardiac death (Yamamoto et al. 2019). Like mice lacking the RRM, mice homozygous for the I536T variant display marked alterations in the splicing of well-established RBM20 target genes but develop neither cardiac dysfunction nor heart failure (Yamamoto et al. 2022). Collectively, these findings suggested that the missplicing of RBM20 target genes alone is not sufficient to explain the severe phenotype in pathogenic *RBM20* variant carriers.

It was not until the first *Rbm20* variant knock-in (KI) animal models were generated that answers were finally provided (Ihara et al. 2020; Schneider et al. 2020). Two variant KI animals were reported simultaneously by different laboratories, each carrying a different genetic variant in a DCM-associated hotspot in exon 9 (c.1881–1920 in humans) (Parikh et al. 2019), which encodes a portion of the arginine/serine-rich (RS) domain in RBM20. Unlike rodents with *Rbm20* ablation, these genetic variant KI animals developed severe DCM with cardiac dysfunction and arrhythmia, even in heterozygous carriers (Ihara et al. 2020; Schneider et al. 2020), mirroring disease in human patients. Critically, these KI animals not only unambiguously demonstrated that pathogenic variants in this hotspot are causative in severe DCM but also brought to light a novel disease paradigm in DCM based on the formation of pathological cytoplasmic RNA granules (Ihara et al. 2020; Schneider et al. 2020). It was subsequently found that these variants disrupt a critical nuclear localization signal located in the RS domain (Zhang et al. 2023a). The presence of cytoplasmic granules has now been confirmed in human patient tissue (Gaertner et al. 2020), as well as in additional animal and human-induced pluripotent stem cell-derived cardiomyocyte (hiPSC-CM) models by our laboratory and others (Wyles et al. 2016; Streckfuss-Bömeke et al. 2017; Briganti et al. 2020; Fenix et al. 2021; Lennermann et al. 2022; Nishiyama et al. 2022; Wang et al. 2022; Zhang et al. 2022; Grosch et al. 2023; Kornienko et al. 2023).

An important question remaining after this discovery is to what extent each of these mechanisms—target gene missplicing and cytoplasmic RBM20 granules—contribute to the pathogenesis of RBM20 cardiomyopathy. For instance, individuals carrying genetic variants in a second DCM-associated hotspot in RBM20 located in exon 11 (c. 2721–2760 in humans) develop DCM (Beqqali et al. 2016; Gaertner et al. 2020; Robyns et al. 2020), yet these variants do not appear to block nuclear import or lead to the formation of cytoplasmic granules like those in the exon 9 hotspot (Gaertner et al. 2020; Zhang et al. 2023a). Instead, limited evidence indicates that exon 11 variants may cause disease through haploinsufficiency that gives rise to missplicing (Beqqali et al. 2016). It remains unclear whether granule formation (unique to exon 9 variants) and haploinsufficiency or missplicing (associated with both exon 9 and 11 variants) lead to phenotypic differences in human patients. However, this distinction has been observed in rodent models, and emerging evidence from studies in hiPSC-CMs suggests that similar differences may also exist in humans. Specifically, 3D-engineered heart tissue models generated with hiPSC-CMs harboring pathogenic *RBM20* KI genetic variants exhibit greater contractile dysfunction than those generated with *RBM20* KO hiPSC-CMs (Fenix et al. 2021).

### Pathogenic RNA granules: a potential common mechanism in cardiac disease driven by localization-disrupting genetic variants in splicing factors

Over a decade ago, pathogenic genetic variants in the *FUS* and *TARDBP* genes, which encode RNA-binding proteins, were linked to the formation of cytoplasmic granules that underlie amyotrophic lateral sclerosis (Kabashi et al. 2008; Kwiatkowski et al. 2009; Vance et al. 2009). The discovery that certain genetic variants in RBM20 also promote the formation of pathogenic granules in the cytoplasm establishes a new paradigm for cardiac disease. An important question is whether this is unique to RBM20 or whether localization-disrupting genetic variants in other RNA-binding proteins, such as AS factors, could also cause cardiac disease through such a mechanism. From a theoretical perspective, it seems likely that this is the case, as many AS factors carry domains/regions capable of supporting the multivalent interactions necessary for granule formation (Lin et al. 2015). Intriguingly, there may already be evidence for another AS factor for which this is the case. A previous study identified a genetic variant in the AS factor Rbfox2 associated with hypoplastic left heart syndrome (HLHS) (Verma et al. 2016), a condition wherein the left ventricle does not form correctly and cannot adequately support systemic circulation. This variant results in premature truncation of the Rbfox2 protein and loss of an NLS at the extreme C terminus of the protein (Verma et al. 2016). Interestingly, such mislocalization may also occur through AS of the *Rbfox2* gene as has been demonstrated in calcific tendons (Cho et al. 2019). Whether these granules are detrimental as they are in RBM20 cardiomyopathy remains to be determined, but this finding provides at least some support that variants in other AS factors may also disrupt heart formation or promote failure through pathogenic cytoplasmic granules.

### Disease-associated variants in splicing factors and their roles beyond splicing

In the following section, we will highlight several AS factors with cardiac disease-associated genetic variants, beginning with RBM20, and their emerging functions beyond splicing. This is not intended to be exhaustive, but instead to point to interesting avenues for exploration of mechanisms that could contribute to cardiac disease alongside missplicing in the context of AS factor genetic variants.

### RBM20

A growing list of nonsplicing functions has been uncovered for RBM20 in recent years. These include the generation of important circular RNAs from the titin gene (Khan et al. 2016), regulation of alternative polyadenylation (Fenix et al. 2021), and the formation of splicing factories (Bertero et al. 2019). It must be mentioned that these nonsplicing functions appear to depend on the splicing function of RBM20, at least to some extent. Namely, ablation of *Rbm20* completely abolishes the production of circular RNAs from the titin gene in mice (Khan et al. 2016). Similarly, there appears to be some degree of coordination between AS and alternative polyadenylation (Zhang et al. 2023b). In the case of splicing factory formation, this also depends on the titin mRNA (Bertero et al. 2019), yet whether transcript splicing is required has not been investigated. Regardless, disruption of these functions has the potential to have far-reaching consequences beyond those attributable to direct missplicing of RBM20's target genes. For instance, it has been shown that while pathogenic variant KI and *RBM20* KO give rise to similar missplicing events in hiPSC-CMs, there are events that are unique in the genetic variant KI cells (Fenix et al. 2021). It is tempting to speculate that this may be due to disruption of events that depend on the RBM20 splicing factories, although other mechanisms cannot be ruled out without additional studies. Therefore, how these functions are impacted by pathogenic genetic variants in the protein warrants further investigation.

### Rbfox family proteins

Members of the Rbfox family of RNA-binding proteins have been shown to play a crucial role in AS events in neuronal and striated muscle, including in the heart (Jin et al. 2003; Underwood et al. 2005; Gallagher et al. 2011). The Rbfox family comprises three members with Rbfox1 and Rbfox2 being expressed in the heart (Jin et al. 2003; Underwood et al. 2005). Exome sequencing of 1213 congenital heart disease probands and their unaffected parents identified three predicted loss-of-function variants in Rbfox2 (Homsy et al. 2015). Interestingly, all three probands, as well as a previously identified proband with a de novo copy number loss encompassing *RBFOX2* identified by the same laboratory (Glessner et al. 2014), manifested HLHS. Dysregulation of Rbfox2 has also been identified in patients with Type 2 diabetes (Nutter et al. 2016). While the pathogenicity of *Rbfox2* variants remains to be validated, knockdown of *Rbfox2* in zebrafish gives rise to an HLHS-like phenotype (Huang et al. 2022). Similarly, conditional ablation of *Rbfox2* in the embryonic mouse heart resulted in the failure to develop normal cardiac chambers (Verma et al. 2022). Current studies have focused largely on the missplicing of Rbfox2 target genes. For instance, *Rbfox2* KO in animal models resulted in aberrant splicing of transcripts coding for mitochondrial, cytoskeletal, and sarcomere components (Huang et al. 2022; Verma et al. 2022). Aside from potential contributions from cytoplasmic granules in the case of variants that disrupt the C-terminal nuclear localization signal (Verma et al. 2016) as discussed above, Rbfox proteins have also been shown to play a role in microRNA biogenesis (Chen et al. 2016). MicroRNAs have emerged as important regulators of cardiac function and disease (van Rooij et al. 2006; Boon and Dimmeler 2015; Zhou et al. 2018). Thus, after establishing the pathogenicity of disease-associated variants, it will be imperative to determine how/whether pathogenic variants affect the processing and maturation of Rbfox-regulated microRNAs, as well as the downstream consequences.

### CELF family proteins

Six different CELF isoforms have been identified with tissue-specific expression patterns that are developmentally regulated (Good et al. 2000; Ladd et al. 2001, 2004). Among CELF isoforms, CELF4 is expressed in multiple tissues, including in the heart (Ladd et al. 2001). Interestingly, a genome-wide association study conducted in childhood cancer survivors identified a single nucleotide polymorphism in CELF4 that was associated with anthracycline-related cardiomyopathy (Wang et al. 2016). This SNP may affect a splice donor site in the CELF4 gene potentially leading to the production of a truncated protein (Wang et al. 2016). CELF proteins are well-known splicing regulators of a developmental transition in the AS of the *TNNT2* gene (Philips et al. 1998; Ladd et al. 2004), which encodes the cardiac isoform of troponin T. Investigation of *TNNT2* splicing in myocardial tissue from patients in this study revealed a significant association between the presence of the SNP and the coexistence of both the embryonic and adult splicing variants of *TNNT2* (Wang et al. 2016). Considering prior findings, it was hypothesized that the continued coexpression of multiple troponin T isoforms enhances cardiotoxicity in response to treatment with high-dose anthracyclines (Wang et al. 2016). Nevertheless, it was recently found that CELF4 plays an important role in translational regulation (Salamon et al. 2023). This is perhaps not surprising given that other members of the CELF protein family are broadly implicated in many facets of RNA biology, including AS, deadenylation, mRNA decay, and translation, among others (Vlasova and Bohjanen 2008; Dasgupta and Ladd 2012). Yet, to what extent disruption of CELF4-mediated translational regulation contributes to predisposition to disease remains an open and intriguing question.

## CONCLUSIONS

The role of AS in cardiac development and disease has garnered increasing attention over the last several decades. The growing identification of genetic variants in AS factors linked to cardiac developmental defects and disease has set the stage for deeper investigations into the underlying molecular mechanisms (Brauch et al. 2009; Johnston et al. 2010; Homsy et al. 2015; Wang et al. 2016; Au et al. 2018; Yu et al. 2018; Chen et al. 2024). Target gene missplicing downstream from pathogenic variants in AS factors is likely to contribute to disease pathogenesis to some extent. Nevertheless, taking a lesson from our own studies on the molecular mechanisms associated with pathogenic variants in RBM20, we advocate for a broader investigation into the contributions of AS factor functions, particularly their nonsplicing roles, to disease pathogenesis. Missplicing may not be the sole mechanism at play. This approach is especially relevant given the expanding roles that many AS factors are now recognized to have in various stages of the RNA life cycle. However, such investigations are not without challenges. Innovative strategies will be necessary to dissect the various nonsplicing functions mediated by AS factors and their contributions to cardiac development and disease.
